# Leveraging Strategic Foresight to Advance Worker Safety, Health, and Well-Being

**DOI:** 10.3390/ijerph18168477

**Published:** 2021-08-11

**Authors:** Jessica M. K. Streit, Sarah A. Felknor, Nicole T. Edwards, John Howard

**Affiliations:** 1 National Institute for Occupational Safety and Health, Cincinnati, OH 45226, USA; 2 National Institute for Occupational Safety and Health, Atlanta, GA 30333, USA; SFelknor@cdc.gov; 3 National Institute for Occupational Safety and Health, Morgantown, WV 26505, USA; NEdwards@cdc.gov; 4 National Institute for Occupational Safety and Health, Washington, DC 20024, USA; JHoward1@cdc.gov

**Keywords:** occupational safety and health, methods and approaches, strategic foresight, forecast, scenario, work-related future, worker well-being

## Abstract

Attending to the ever-expanding list of factors impacting work, the workplace, and the workforce will require innovative methods and approaches for occupational safety and health (OSH) research and practice. This paper explores strategic foresight as a tool that can enhance OSH capacity to anticipate, and even shape, the future as it pertains to work. Equal parts science and art, strategic foresight includes the development and analysis of plausible alternative futures as inputs to strategic plans and actions. Here, we review several published foresight approaches and examples of work-related futures scenarios. We also present a working foresight framework tailored for OSH and offer recommendations for next steps to incorporate strategic foresight into research and practice in order to advance worker safety, health, and well-being.

## 1. Introduction

In recent decades, factors affecting worker safety, health, and well-being in advanced industrialized countries like the United States have undergone a fundamental shift. Today, numerous social, technological, economic, environmental, and political (STEEP) trends demonstrate complex patterns of influence on work, the workplace, and the workforce [1,2,3,4,5,6,7]. Because the number of relevant trends is too vast to comprehensively address in this paper, examples representing the different categories of STEEP are offered here. Rapid market shifts and advances in technology have contributed to a dramatic rise in part-time, temporary, contract, on-call, contingent, and ‘gig’ work. While these nonstandard work arrangements may increase work-life flexibility, they also tend to leave workers at risk by offering fewer protections and comparatively lower rates of pay [8,9,10]. Workplace automation has created new jobs and improved job safety in certain industries while concurrently contributing to widespread work intensification, job displacement, and wage reductions [11,12,13]. Demographic shifts and environmental changes, including climate change, have resulted in a highly diverse labor force with varying wants, needs, lifestyles, and vulnerabilities, which further exacerbate the effects of other STEEP trends [14,15,16,17,18]. Furthermore, since 2020, the COVID-19 pandemic has demonstrated how quickly a lack of preparation for events such as public health crises can accelerate changes that transform entire industries and have a compounding effect on worker safety, health, and well-being [19,20].

The occupational safety and health (OSH) community has already acknowledged the need for a transformation to keep up with the pace of the rapid and profound changes affecting workers [2,4,5,21,22,23]. As part of that transformation, an approach for expanding the focus of OSH was proposed in 2019 to more fully consider how traditional job risks and hazards combine with personal, social, and economic factors to affect health and well-being across the working life continuum [5]. Proactively managing this broader—and growing—list of issues will require innovative and systems-focused OSH methods and approaches [2,4,5,21,22]. To that end, this paper proposes integrating *strategic foresight* into OSH research and practice. This future-oriented way of thinking and planning can help OSH professionals more actively anticipate, and even shape, the systems influencing the future of worker safety, health, and well-being.

### 1.1. A Brief Overview of Strategic Foresight

Strategic foresight is a practice rooted in futures studies that is designed to help better understand, prepare for, and influence the future [24]. At its core, strategic foresight recognizes that the future is not predetermined or predictable [25]. Instead, the roots of multiple plausible futures exist today in the form of weak or early signals of potential change [26]. Identifying and monitoring these signals can reduce the likelihood of being unprepared for or surprised by emerging trends and changes as they arrive in the mainstream. It can also uncover points at which today’s decisions and actions can be leveraged to move toward desirable futures.

Engaging in strategic foresight involves the completion of two distinct, yet interrelated, tasks. Because the future is not preordained, the first task includes mapping futures, or developing functional views of alternative futures that are sometimes referred to as *forecasts* by strategic foresight practitioners in the United States [27,28,29]. These functional futures are the product of the systematic observation, organization, and synthesis of the weak and early signals of change found in the present. They are not intended to be accurate predictions of the future. Rather, they are designed to be provocative but realistic visions of what the future could reasonably be, based on the signals of change that exist today. For the second task, the implications and critical issues associated with the alternative functional futures are assessed to influence the future by informing the design and implementation of feasible and responsive strategic options [26].

It is important to note that strategic foresight is a complement to, not a substitute for, strategic planning. Traditional strategic planning reviews evidence from the past and asks how we might do things better, faster, or more proficiently in the future. This backward-to-the-future approach is very useful and efficient in stable and unchanging environments [30]. Conversely, strategic foresight looks ahead and asks what *may* be coming, how it *might* affect us, and what we *can* do today to start moving toward a preferred outcome. This forward-facing perspective is particularly useful during periods of complex instability, the conditions of which are often described as being volatile, uncertain, chaotic, and ambiguous (VUCA) or turbulent, uncertain, novel, and ambiguous (TUNA) [30,31].

### 1.2. Approaches to Strategic Foresight

Many different models and frameworks have been created to facilitate the application of strategic foresight as part of planning efforts, often in response to VUCA or TUNA conditions [32]. Some of the formalized approaches frequently cited in the published literature are presented in Table 1. Other approaches, such as the Institute for the Future’s copyrighted *Foresight Toolkit* and the digital Futures Platform, are available via direct distribution for a nominal fee [33,34]. Though there may be advocates for one approach over another, it is important to note that the relative effectiveness and utility of the various approaches have not been directly compared, and there is no clear evidence to suggest that one strategic foresight approach is superior to any other. Instead, practitioners typically select an approach based on its fit with critical contextual factors, such as past and current conditions and the needs and interests of key stakeholders [35].

Though the popular models and frameworks each have a slightly different approach to strategic foresight, they all include steps that align with the two main activities of strategic foresight: (1) Generating alternative futures; and (2) assessing implications to inform decisions and actions. They also share two other important features. First, each approach conceptualizes time in terms of near-, mid-, and far-term horizons. Second, they all produce alternative futures in the form of scenarios. These two critical strategic foresight concepts—*time horizons* and *scenarios*—are described here in greater detail.

#### 1.2.1. Time Horizons

**Figure 1:**
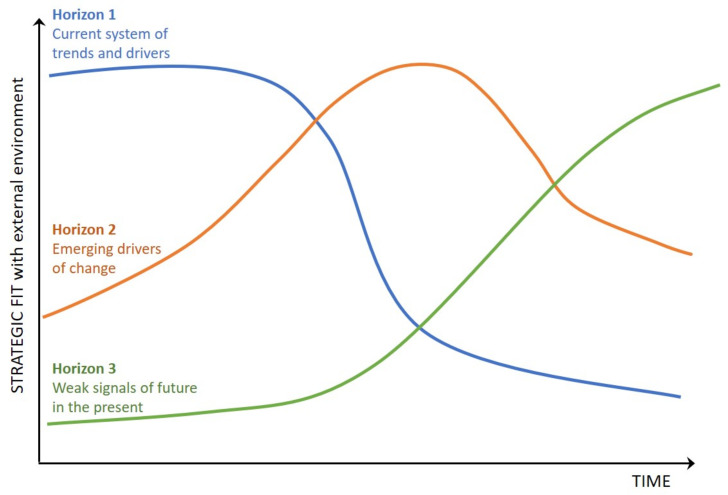
Visual representation of Three-Horizon Foresight [43].

A flexible method commonly used to delineate the near-, mid-, and far-term futures is Three-Horizon Foresight, which is typically depicted with a schematic similar to Figure 1 [43,44]. As its name implies, Three-Horizon Foresight divides time into three horizons. Horizon 1 is the current prevailing system, or way of doing things. As time moves forward and change inevitably occurs, the Horizon 1 system becomes increasingly less likely to align with the challenges and opportunities found in the STEEP environment. In contrast, Horizon 3 represents marginal ideas and arguments falling outside the current prevailing system, hints of which are seen as weak or early signals in the present. Over time, these early signals may increase in both strength and strategic fit with the changing STEEP environment and eventually become mainstream in a new prevailing system. Horizon 2 is the period of transition between Horizon 1, the near-term state, and Horizon 3, the far-term state.

While it is common to define Horizon 3 (the far-term future) as 10 to 20 years from the present, this is not a hard-and-fast rule [45]. The amount of time accounted for by each Horizon should be determined by the most useful planning or business cycles that are relevant to the focal topic and its key stakeholders [35,43]. For topics with a history of being slow to change (e.g., cultural norms, environmental climate), foresight practitioners are more likely to define longer horizons because it takes more time for change to occur and manifest in the mainstream. For projects investigating topics that experience change more rapidly (e.g., technological innovations), or those tied to faster-paced business cycles, time horizons may be shorter.

#### 1.2.2. Scenarios

A *scenario* is a story with a carefully constructed plotline describing one plausible future [39,46,47]. Because the future is not predetermined or predictable, multiple future visions are usually articulated as separate scenarios during a strategic foresight project. Exactly how many scenarios should be produced will vary based on end-user needs and the scenario development techniques used, the latter of which are discussed later in this paper. However, published recommendations typically range from two to six scenarios per foresight project [48]. More important than the number of scenarios produced is ensuring that each one represents a unique, plausible, and logical story offering new insights into future possibilities, threats, and opportunities based on careful analysis of inputs [49,50].

There is no one right or best way to communicate alternative futures scenarios. In practice, a variety of formats and products can be used to describe different scenarios [39,51]. Some common options are identified in Table 2.

### 1.3. Uses of Scenario-Based Strategic Foresight

Scenarios have long been utilized as part of business, military, public policy, and emergency preparedness strategic planning efforts [50,56,57,58]. The practice originated in the 1950s, when the RAND Corporation first began using scenario techniques to develop U.S. military strategies [59]. Around the same time, the Centre d’Etudes Prospectives was established to develop scenarios of possible political, social, and cultural futures for France [60]. In the 1960s, futures groups such as the Institute of the Future, the Stanford Research Institute’s (SRI) ‘Futures Group,’ and the California Institute of Technology began using scenarios as a public policy planning tool [56]. Soon after, Royal Dutch Shell permanently adopted scenario planning as part of its business strategy. Through its ‘Year 2000 Project,’ the company developed scenarios that depicted looming oil scarcities and price increases, which helped Royal Dutch Shell prepare for major adverse events in the oil industry, such as the 1973 oil crisis and the 1980s oil bust, which resulted in a competitive advantage during those times of turbulence [61]. Following the success of Royal Dutch Shell’s scenario planning efforts, major organizations across a variety of industries began to incorporate scenario planning into their business culture. Examples include Motorola, General Electric, United Parcel Service, Philips Corporation, Nokia Corporation, Siemens, and Daimler AG [58]. Using scenarios as a planning tool has allowed these and many other organizations to remain resilient through—and even thrive on—the many changes and challenges they have faced over time [62].

The current widespread use of strategic foresight to anticipate future uncertainties across a variety of domains is well-documented in the published literature. Numerous private and public sector organizations around the world report regularly engaging in the practice to gather and make sense of information about the future contexts in which they will operate [63,64]. Today, a growing worldwide community of organizations engages in scenario-based strategic foresight to explore the implications of plausible future changes to work, the workplace, and the workforce. A list of some of the most active organizations in this space culled from a comprehensive search is presented in Table 3. The search exhausted combinations of keywords from three categories: Organization type (organization/organization, center/centre, council, foundation, initiative, institute, lab, office, program/programme, work group, workgroup), foresight activities (strategic foresight, changing patterns, forecasting, foresight, future scenarios, horizons, horizon scanning, scenario), and work futures orientation (future of work, changing nature of work, digital, fourth/4th industrial revolution, future of employment, future of jobs, future workplace, industry 4.0). Though the organizations uncovered by this search are primarily headquartered in North America and Europe, OSH-related foresight initiatives are growing in other areas of the world as well. Examples include the Institute for Futures Research at Stellenbosch University in South Africa and the Ajman Department of Economic Development’s “Future Foresight Initiative” in the United Arab Emirates, announced in 2020 [65,66].

The remaining sections of this paper explore how strategic foresight has been and could be applied to anticipate future challenges and opportunities in OSH that may affect worker safety, health, and well-being. First, we review two popular methods for constructing plausible future scenarios and demonstrate how each has been used to explicate possible work-related futures and their potential risks and hazards (Section 2. Developing Plausible Future Scenarios for Worker Safety, Health, and Well-Being). Next, we highlight several examples showing how strategic foresight has been used to develop recommendations and policy options that protect and promote worker safety, health, and well-being (Section 3. Using Scenarios to Protect and Promote Worker Safety, Health, and Well-Being). Then, we describe a foresight framework currently being tailored for the OSH community (Section 4. Foresight Framework for Occupational Safety and Health (OSH)). Finally, we discuss future directions and recommendations for applying strategic foresight to OSH research and practice (Section 5. Conclusions and Recommendations).

## 2. Developing Plausible Future Scenarios for Worker Safety, Health, and Well-Being

Constructing scenarios as part of a strategic foresight effort is, arguably, equal parts science and art [67]. A variety of techniques and methods exist to aid and guide the scenario development process. This section offers a deeper look into two popular methods that are frequently used to construct alternative work futures scenarios: The matrix method and the archetypes method.

### 2.1. The Matrix Method of Constructing Future Scenarios

The matrix method, also known as the 2 × 2 double uncertainty method, is one of the most widely used scenario building techniques in advanced industrialized nations like the United States [51,68]. The method rose in popularity after Royal Dutch Shell’s success in using scenario-based planning to prepare for changes in mid- to long-range global energy demands and costs [69]. An overview of the matrix method is also included as an appendix in *The art of the long view: Planning for the future in an uncertain world*, considered by many as the seminal publication on scenario-based planning [62].

The matrix method begins with the identification of two high-impact, high-uncertainty issues that will influence the future for a domain of interest [45]. The issues then become the perpendicular axes of a 2 × 2 matrix, and the poles of each axis are defined. The result is a grid of four quadrants, into which scenarios of alternative futures for the focal domain can be mapped.

### 2.2. Applying the Matrix Method to Develop Alternative Work Scenarios

The following examples demonstrate the use of the matrix method to construct scenarios describing alternative futures and their potential effects to worker safety, health, and well-being.

The European Agency for Safety and Health at Work (EU-OSHA) used the matrix method to explore potential future OSH risks associated with digitalization [70]. The resulting four futures, presented in Table 4, are driven by the key uncertainties of economic growth and technology application (supportive vs. resistive) and governance and public attitude (low vs. high). Work-related risks shared across the scenarios include (1) the use of technologies—particularly automation—eliminating some known job hazards while also introducing new ones; (2) changes to work flexibility, work pace, and work management; (3) alternative business and employment models; (4) increased job instability; (5) loss of privacy to surveillance; and (6) increased sedentariness.

PricewaterhouseCoopers (PwC) espoused four plausible futures for the 2030 workforce [71]. These futures, built around the dichotomies of individualism vs. collectivism and corporate integration vs. business fragmentation, are depicted in Table 5. Together, these four futures shed light on possible long-range challenges for workforce selection and recruitment, performance and personnel management systems, and learning and development programs. They also highlight the staying power of automation as a megatrend affecting worker safety, health, and well-being.

The World Economic Forum (WEF) extended the 2 × 2 method and included a third uncertainty, yielding a 2 × 2 × 2 matrix describing eight alternative scenarios of work in 2030 [72]. These are described briefly in Table 6. The rate of technological change (steady vs. accelerating), the evolution of learning (slow vs. fast), and talent mobility (low vs. high) served as the key uncertainties around which the scenarios were constructed in order to identify strategic options that can begin proactively shaping a better future for workers.

### 2.3. The Archetypes Method of Constructing Future Scenarios

Like the 2x2 matrix method, the archetypes method of scenario building also results in the development of up to four plausible alternative futures. Rather than being constructed around two polarized key drivers, however, the futures are constructed using a larger set of drivers and prototypical archetypes that describe common patterns of change. There are a number of potential archetype sets that can be applied to generate futures scenarios [73]. One of the most popular is a set of four archetypes, which are the product of the Hawaii Research Center on Futures Studies’ extensive cross-cultural research on images of the future [41,74]. Over time, multiple well-known foresight practitioners have genericized the definitions of these four popular archetypes to facilitate the exploration of the future for a variety of topics [75,76]. These broader archetype definitions include:

- *Continuation* (or *Continued Growth*): A future where the trends of the present accelerate without any major changes or disruptions.
- *Collapse*: A future where the current system fails due to some negative force(s) or dysfunction.
- *New Equilibrium* (or *Constraint*): A future where the current system is challenged in some way and must respond with some type of change to achieve a new sense of balance and stability.
- *Transformation*: A future where there is a fundamental change and the current system is discarded for an entirely new one.

### 2.4. Applying the Archetypes Method to Develop Future Work Scenarios

The futures constructed using the archetypes method will vary based on the focal domain and selection of key issues and drivers of change. Two examples are presented here to demonstrate the potential diversity of the resultant scenarios.

Using 15 global challenges ranging from climate change and public health to energy and technology, The Millennium Project Team developed three plausible futures for work and technology by 2050 [77,78]. In the “It’s Complicated—A Mixed Bag” continuation scenario, an accelerated use of technology, high rates of unemployment, and the mixed success of a universal basic income yield a multipolar world where large corporations have more power and control than the government. In the “Political/Economic Turmoil—Future Despair” collapse scenario, an unanticipated explosion of unemployment in the 2030s leads to political turmoil, terrorism, and high rates of organized crime by 2050. In the “If Humans Were Free—The Self-Actualization Economy” transformation scenario, governments are able to achieve a self-actualized, steady-state economy by adequately preparing for the effects of artificial intelligence in the workplace, researching methods to phase in universal basic income, and promoting self-employment.

In another example, the Organisation for Economic Co-operation and Development (OECD) generated three different plausible archetypal futures by focusing on potential directions for national policies to support economic growth and job development [53]. In the “Quick Fixes” continuation scenario, policymakers attempt to address the inequalities created by technological advances, nonstandard arrangements, and high unemployment rates with redistribution strategies, such as high wealth taxation rates. In the “Multipolar” constraint scenario, low interest rates, private credit programs, and education systems built around lifelong learning allow governmental policy to focus on the creation of new jobs and industries and the regulation of technology—in particular, augmentation—to prevent the creation of new inequalities. In the “City Power” transformation scenario, technologies are used to address climate and food scarcity issues and support collaboration between cities, businesses, and governments while national-level policies are enacted to regulate nonstandard work arrangements, leverage the use of private data for public good, and develop social good metrics.

## 3. Using Scenarios to Protect and Promote Worker Safety, Health, and Well-Being

Well-constructed scenarios can present interesting, and sometimes entertaining, views of the future. However, their real value lies in their ability to spark strategic conversation and action [62]. As previously noted, the strategic foresight process is not complete after the development of scenarios. Instead, a comprehensive strategic foresight project should also support the creation of strategies that help individuals and organizations prepare for a range of plausible alternatives and move toward a preferred future outcome [41,79].

Some published scenarios related to the future of work have been developed for the sole purpose of spurring additional dialogue about the current state of preparedness for potential future conditions. These scenarios do not result in the development of specific strategic options and plans for stakeholders. Instead, they are analyzed to a limited extent to shed light on key issues and the need for strategies to address them. The scenarios from EU-OSHA, PwC, and OECD described in Section 2 were constructed for this purpose [53,70,71].

In other instances, scenarios describing plausible futures related to work have been analyzed not only to identify key themes and challenges, but also to yield specific and feasible recommendations for stakeholder action [68]. This additional layer of analysis typically involves several steps: (1) Establishing a vision and goals for the future, (2) examining multiple scenarios to identify common opportunities and threats, (3) creating a timeline of viable actions that will aid in accomplishing the established goals within the context of the opportunities and threats, and (4) pinpointing key decisions and resources required for each action [80]. WEF, for example, analyzed the eight scenarios for the future of work by 2030 presented in Table 6 to generate recommendations for collaborative action by governments, businesses, and academia. These include workforce reskilling and education reform; expanded access to communication technologies; increased incentives and support for job creation and protection, labor force participation, and entrepreneurship; enhanced oversight and management of platform work and talent mobility; and the development of agile and sustainable social safety nets [72]. Similarly, The Millennium Project Team analyzed its three archetype scenarios described in Section 2.4 to identify over 90 possible stakeholder actions with accompanying implementation guidance in support of creating an equitable, humanitarian future economy. These actions were clustered into five major groupings: Government and governance; business and labor; science and technology; education and learning; and culture, arts, and media [77]. Other scenario-based studies have yielded similar results, generating action and policy recommendations for specific OSH topics, industry sectors, and geographic regions [19,81,82,83]. Though the publication of follow-on impact studies is rare, the available evidence suggests future of work scenarios and recommendations generated by the application of strategic foresight can be used to inform the development of government initiatives; increase subject matter knowledge among stakeholders and the general public; enhance professional education, training, and development offerings; and expand foresight awareness and capacity [82].

## 4. Foresight Framework for Occupational Safety and Health (OSH)

The concept of applying strategic foresight to the exploration of work-related futures is not entirely new. A number of scenarios have been published describing potential futures with respect to work [68]. However, compared to its use in other business planning and decision-making efforts, strategic foresight has been arguably underutilized for OSH. This may be due, at least in part, to the fact that the majority of foresight research and practice lives within futures studies and technology studies, which have not historically been considered OSH disciplines. Consequently, strategic foresight experts often tend to publish on topics outside those central to OSH in outlets beyond those on which the OSH community most often relies for new information [84].

**Figure 2:**
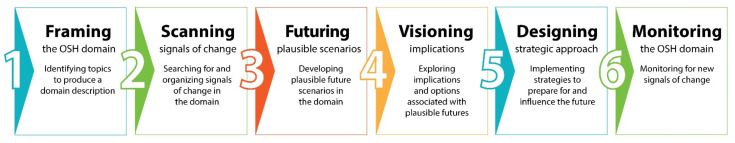
Foresight Framework for Occupational Safety and Health (OSH). Adapted from the UH Foresight Framework [85,86].

To further the application of strategic foresight in the OSH domain, the Centers for Disease Control (CDC) National Institute for Occupational Safety and Health (NIOSH) has adapted the widely used University of Houston Framework Foresight [28,85,86]. NIOSH elected to build on Framework Foresight because it is an internationally renowned approach that is versatile and flexible enough to accommodate a variety of project topics and aims while also providing a clear, step-by-step roadmap through the foresight process. The intent of this NIOSH Foresight Framework for OSH, presented in Figure 2, is to help bridge the current OSH-foresight gap and bring foresight into OSH conversations and planning practices.

Like the University of Houston Framework Foresight, the NIOSH Foresight Framework for OSH has six discrete stages that are interrelated and interdependent. The first stage of *Framing* the OSH domain involves identifying the domain or topic of interest and developing a description of the domain, including the central question or issue to be explored, the ‘client’ or intended audience of the foresight effort, the geographic scope of the domain to be explored, and the time horizons the project will consider. It is important to devote ample time to the *Framing* stage to ensure the domain, central question, client and client needs, scope, and time horizons are adequately and accurately defined, as these factors largely influence the activities completed in the subsequent framework stages. The second stage, *Scanning*, is central to the foresight effort and involves searching for information about how things might be different in the future. During this stage, practitioners look for and organize signals of change within the domain of interest and the broader STEEP context. A variety of information sources should be reviewed during the scan, not only including refereed publications and major surveillance systems, but also government and stakeholder reports, legislative records, trade and technical journals, newsletters, monographs, blogs, mainstream and fringe media, general internet searches, and individuals with a variety of perspectives on the domain topic [86]. The information gathered is used to construct key drivers of the future for the selected domain. The third stage, *Futuring*, involves developing alternative future scenarios from the drivers using techniques that meet the needs of the identified client. Common techniques will likely include the four scenario archetypes method or the 2x2 matrix method described in Section 2. In the fourth stage, *Visioning*, the implications of the different scenarios are considered for the client. Assessing implications can uncover potential risks, challenges, and opportunities associated with each scenario and identify the client’s degree of preparedness for implementing the changes needed to create and sustain the client’s preferred future. The fifth stage of *Designing* involves planning and constructing strategic approaches that can guide the client’s actions today in support of the desired future. The last stage of *Monitoring* involves continuing to scan for new signals of change and updating the domain topic as needed to further refine future foresight efforts. Engagement with and integration of relevant stakeholders is encouraged throughout the entire foresight process and should be tailored at each stage to effectively support the project purpose.

Though the framework is presented as a sequential model, the strategic foresight process is not entirely linear. Both during and at the end of each stage within the framework, users are encouraged to reflect upon the activities they have completed in previous stages and determine if any additional work is needed before moving on the to the next stage. Signals of change detected in the *Scanning* stage, for example, may highlight the need to revise the domain description, central question or issue, or geographic scope defined during the *Framing* stage. Similarly, attempts to develop alternative scenarios during the *Futuring* stage may uncover the need to revisit *Scanning* to search for additional signals of change to create provocative but realistic stories for what the future could entail. In addition, the sixth stage of *Monitoring*, which involves continued scanning for signals of change over time, is an inherent extension of the second stage of *Scanning*, which involves a time-bound, dedicated initial scan for signals of change as part of a foresight project. Over time, signals uncovered during *Monitoring* may suggest the need to revisit *Framing* to redefine the domain or to re-enter the *Futuring* stage to update the plausible scenarios for a given domain.

To test this framework, NIOSH has designed a foresight pilot project exploring “the future of OSH” as a priority domain. NIOSH has convened a diverse team of subject matter experts from across the OSH discipline to participate in this pilot test of the framework. This approach aligns with recommendations of bringing together multidisciplinary research teams when using scenario-based strategic foresight to explore complex issues and topics [87]. The project was currently underway at the time this paper was written, and the project team, led by authors of this paper, plan to report the process and results of the effort in a future peer-reviewed publication. In addition, at the time this paper was written, NIOSH had formal agreements in place with international strategic foresight experts to evaluate the utility of the Foresight Framework for OSH. It is anticipated that the results and lessons learned from the pilot test and evaluation will inform the development of strategic options and recommendations for NIOSH’s future research and service activities and contribute to the refinement of the NIOSH Foresight Framework for OSH.

## 5. Conclusions and Recommendations

The world of work today is not what it was ten, five, or even two years ago. Socioeconomic, geopolitical, demographic, and technological megatrends are anticipated to have disruptive influences on the future of work [88]. Exactly how these trends will change and unfold over time is largely unknowable, and their potential future impacts to workers are unclear. Because of VUCA/TUNA conditions, the OSH community cannot sufficiently identify and prepare for the potential future risks and hazards that may influence worker safety, health, and well-being using only conventional strategic planning. Complementary forward-looking methods are also needed to help us design and refine proactive risk management programs and strategies for the future of work before it arrives.

Strategic foresight is a tool the OSH community can leverage to bring futures thinking into research and practice. Generating and assessing the implications of multiple plausible work-related futures can help OSH prepare for, plan, and influence the future by combatting two critical errors of decision making: The human tendencies to over- and underpredict change [50]. Strategic foresight can expand the range of possibilities OSH envisions for work, the workplace, and the workforce of the future while ensuring those visions remain grounded in reality. This broader consideration of possible, yet realistic, future conditions can provide insights into environmental and market changes; facilitate meaningful strategic conversations; and aid in the identification of opportunities and threats, the reduction and management of uncertainties, the coordination of objectives, and the consideration and adoption of alternative perspectives [89].

Existing OSH research initiatives and programs, such as the NIOSH Future of Work Initiative and *Total Worker Health*^®^ Program, can provide pathways to prospective domains to explore using strategic foresight. The NIOSH Future of Work Initiative, for example, has developed a framework to guide research and practice-based activities relevant to the future of work, the workplace, and the workforce in the United States [6]. The nine central priority topics include organizational design, technological job displacement, work arrangements, artificial intelligence, robotics, technologies, worker demographics, economic security, and skills. The framework also recognizes additional high-level issues, including globalization, extreme weather conditions, and emergency and disaster preparedness and response for situations such as the COVID-19 pandemic, that have a widespread impact on work, the workplace, and the workforce. Collectively, these priorities can help focus inquiry into plausible future scenarios related to work. Similarly, the NIOSH *Total Worker Health^®^* Program has published an extensive list of key issues relevant to advancing worker well-being [5,23]. These issues are associated with the organization of work, built environment, leadership, compensation and benefits, community, workforce demographics, policy, and new employment patterns. These issues, as well as many others identified throughout NIOSH’s entire Program Portfolio, which is organized by industrial sectors and health and safety outcomes, could be used to help focus future strategic foresight efforts within the OSH domain [90].

To ensure strategic foresight has staying power in the OSH community, it will be imperative to invest time and resources into building strategic foresight capacity. Enhancing OSH awareness and knowledge of strategic foresight will be key. The Foresight Competency Model (FCM) developed by the international Association of Professional Futurists (APF) can serve as a guide for enhancing foresight knowledge, skills, and abilities in OSH. The FCM identifies six core competencies for foresight practitioners—framing, scanning, futuring, designing, visioning, and adapting—which align 1:1 with the steps of the University of Houston Framework Foresight model and, subsequently, NIOSH’s adapted Foresight Framework for OSH [28,85,91]. APF also offers a prototypical plan for developing these six competencies in individuals, individual team members, and entire teams [91]. Mentoring and training opportunities will be central to competency development for all three groups. To this end, a number of internationally recognized foresight organizations in the United States (e.g., the University of Houston and the Institute for the Future) and Europe (e.g., Saïd Business School at the University of Oxford and Copenhagen Institute for Futures Studies) offer professional foresight training programs and consulting for a nominal fee [33,92,93,94]. A number of foresight guidance documents and recorded presentations are also available online, free of charge [30,95,96,97]. Building formal and informal partnerships with entities already active in strategic foresight can serve as a complement to structured learning. Table 3 provides a reasonable starting list of potential partner organizations, though they do not represent the entire universe of viable strategic foresight partners for OSH. The Federal Foresight Community of Interest (FFCOI), for example, was established in 2013 as an interagency organization to provide a forum for federal agencies in the United States interested in applying foresight [98]. Though many of FFCOI’s participating agencies may not practice foresight in the OSH space, FFCOI is well-positioned to provide OSH with insights on foresight best practices and strategies for building and sustaining a connected community of foresight practitioners.

The future may be largely unpredictable, but it does not have to be a complete surprise when it arrives. Clues of what the future may hold exist today in the form of weak and early signals of change. The practice of strategic foresight empowers individuals and organizations to leverage those signals to gain realistic insights into the future and begin developing plans and options to move forward in preferred directions. Applied to OSH, strategic foresight may help inform the development of proactive systems to prevent injury, illness, death, and disability and promote worker well-being across the working life continuum for generations of future workers.

## Figures and Tables

**Table 1:** Popular formalized approaches to strategic foresight described in the published literature.

Approach	Originating Organization	Overview of Steps (with *Step Name* Included, Where Applicable)
Assumption-based planning [36,37]	The RAND Corporation	• Identify assumptions underlying current operations or plans • Identify assumptions that may be vulnerable to or violated by future changes • Define signposts, or events that would indicate an assumption is becoming more or less vulnerable over time • Define shaping actions that will either cause or prevent the failure of a vulnerable assumption • Define hedging actions that can help better prepare for a potential assumption failure in the future
FORLEAN [38]	European Commission	• *Diagnosis:* Reflect on the current system • *Exploration:* Build scenarios of possible evolutions of the system • *Strategic Orientation:* Discuss possible strategies • *Making Choices:* Encourage open debate to reach consensus • *Implementation and Coordination:* Translate findings into action
Framework Foresight [28,39]	University of Houston	• *Framing:* Identify the domain, or boundaries and key categories of what will be explored • *Scanning:* Scan the internal and external environments for information and trends related to the domain • *Forecasting:* Identify drivers and uncertainties, then create alternative futures • *Visioning:* Identify implications, challenge assumptions, and develop a strategic vision • *Planning:* Develop strategic options • *Acting:* Communicate results, create an action plan, and institutionalize strategic thinking
Generic Foresight Process [40]	Swinburne University of Technology	• *Inputs:* Scan the external environment to identify changes that are shaping the future • *Analysis:* Analyze the scanning results to explore potential shifts needed to identify strategic implications • *Interpretation:* Identify assumptions and worldviews affecting how the future is interpreted • *Prospection:* Develop alternative images, or scenarios, for plausible and preferred futures • *Outputs:* Identify strategic options • *Strategy:* Agree on action to take today
Manoa Futures Visioning Process [41]	University of Hawaii	• *Appreciate the Past:* Explore the history of the community or group involved • *Understand the Present:* Discuss the problems and possibilities of the present • *Forecast Aspects of the Future:* Discuss possible challenges and opportunities from the futures • *Experience Alternative Futures:* Craft alternative futures trends, emerging issues, challenges, and opportunities from the future • *Envision the Futures:* Envision a preferred future • *Create the Futures:* Decide the sequence of what to do now to move toward the preferred future • *Institutionalize Futures Research:* Set up an ongoing ‘futures’ unit to keep the process going
Oxford Scenario Planning Approach [31]	University of Oxford	• Develop an understanding of the problematic situation • Define, develop, verify, and refine a set of strategic frames—the underlying structures of belief, perception, and appreciation used to make sense of the world • Generate alternative scenarios • Engage in iterative learning cycles comprised of: • *Reframing:* Contrasting alternative scenarios of the future to reveal, test, and redefine the official future, generate alternatives, and generate new knowledge and insights • *Reperception:* Defining a new course of action
Exploring the Future [42]	Royal Dutch Shell	• *Preparation:* Assembling a clear description of the project, goals, and resources • *Pioneering:* Challenging assumptions and identifying themes • *Map-making:* Building and vetting the scenarios • *Navigation:* Presenting the scenarios to inform plans and actions of key stakeholders, and refining the scenarios based on feedback • *Reconnaissance:* Examining implications, interpreting signals of change, and further disseminating the scenarios • *Preparation:* Beginning the process anew to develop fresh scenarios in response to change over time

**Table 2:** Common products used to communicate alternative futures scenarios.

Product Type *	Definition
Artifact	Linguistic, conceptual, cultural, and material objects and articles, including physical tools, technical processes or procedures, or symbols and logos [52]
Headline or News Story	Brief captions or stories describing events that may happen in the future [53]
Narrative	Stories of organizations and the people in them that rethink the past, reconsider present conditions, and reimagine the future [54]
Persona	Characters who live in one plausible future and fully embody the human representation in that future [55]

* Multiple products can be used to convey a single scenario.

**Table 3:** Organizations using strategic foresight to create and explore plausible future changes to work, the workplace, and the workforce.

Headquarters Location	Organization Name	Organization Type *
Australia	Institute for Safety, Compensation, and Recovery Research	Research
Belgium	European Parliamentary Research Service	Government
Canada	Brookfield Institute for Innovation + Entrepreneurship	Research
	Centre for International Governance Innovation	Think Tank
	Institut National de la Recherche Scientifique (INRS)	Academic
	Policy Horizons Canada	Government
Finland	Demos Helsinki	Think Tank
France	Organisation for Economic Co-Operation and Development	IGO
Germany	Federal Ministry of Labour and Social Affairs	Government
	Fraunhofer-Gesellschaft	Research
Ireland	European Foundation for the Improvement of Living and Working Conditions (Eurofound)	IGO
Netherlands	Netherlands Organisation for Applied Scientific Research (TNO)	Research
Scotland	Scotland’s Futures Forum	Think Tank
Spain	European Agency for Safety and Health at Work (EU-OSHA)	IGO
Switzerland	International Labour Organisation (ILO)	IGO
	World Economic Forum (WEF)	NGO
United Kingdom	Deloitte	Consulting
	Health and Safety Executive	Government
	PricewaterhouseCoopers (PwC)	Consulting
	Rethinkery Foresight	Consulting
	The Royal Society for Arts, Manufactures and Commerce (RSA)	Research
	Schumacher Institute	Think Tank
	University of Oxford, Saïd Business School	Academic
United States	Cognizant	Consulting
	Data & Society Research Institute	Research
	Future-IQ	Consulting
	The Institute for the Future	Think Tank
	International Association of Outsourcing Professionals	Consulting
	McKinsey Global Institute	Consulting
	Millennium Project	Think Tank
	RAND Corporation	Research
	Toffler Associates	Consulting

* IGO = Intergovernmental Organization; NGO = Non-Governmental Organization.

**Table 4:** EU-OSHA’s four futures of occupational safety and health risks from digitalization [70].

	Low economic growth and technology application	High economic growth and technology application
Supportive governance and public attitude	**Evolution** Technology significantly changes half of all jobs. There is continued investment in OSH to address dangerous and unhealthy work.	**Transformation** Technology significantly impacts most jobs. At the same time, work safety and quality remain a high priority.
Resistive governance and public attitude	**Fragmentation** Technology has had a low impact on jobs overall, though many low-skill repetitive jobs have been fully automated.	**Exploitation** Technology use varies by industry. Routine, repetitive jobs have been fully automated to save costs. Job competition is high.

**Table 5:** PwC’s four futures for the 2030 workforce [71].

	Individualism	Collectivism
Corporate Integration	**Blue World** Capitalism rules, widening the wage gap. Performance-enhancing augmentation technologies, medications, and implants are normalized. Privacy is lost to continuous employer surveillance inside and outside the workplace.	**Green World** Corporate social responsibility rules. Employers offer fair pay, family-friendly policies, and skills development. The increased use of technology for ethical and environmental reasons reduces the number of available jobs.
Business Fragmentation	**Red World** Innovation rules. High rates of technology use decrease job opportunities, inflate market pressures, and increase work pace. Skills, not workers, are valued.	**Yellow World** Business ethics rules. A collective desire for the fair distribution of wealth and resources drives policy. Autonomous and flexible work provides purpose and fulfillment.

**Table 6:** WEF’s eight futures of work by 2030 [72].

	Steady Technological Change		Accelerated Technological Change	
	Low Talent Mobility	High Talent Mobility	Low Talent Mobility	High Talent Mobility
Slow Learning Evolution	**Workforce** **Autarkies** A large number of displaced workers compete for few jobs. Governmental policies restrict international labor mobility.	**Mass Movement** Worker mobility has resulted in steady incomes, lower living costs, and high levels of competition between workers at all skill levels.	**Robot Replacement** Widening skills gaps have increased inequalities and polarized views. Borders are tightly controlled in an effort to keep talent local.	**Polarized World** Due to fast-paced tech and low-paced learning, large portions of the workforce are unemployable. ‘Super economies’ of high-skilled people trade only with one another.
Fast Learning Evolution	**Empowered** **Entrepreneurs** Lifelong learning is embraced. Workers are able to create their own opportunities in dynamic markets, but migration is restricted in an attempt to retain talent.	**Skilled Flows** A fast-paced skills evolution enhances creativity and productivity. Abundant opportunities normalize labor mobility. Inequality at the country level increases based on access to tech resources.	**Productive Locals** There is high demand for workers to complement machines. Borders are tightly controlled in an effort to keep talent local.	**Agile Adapters** The global workforce is mobile and agile. There is worldwide harmony of social and workforce policies, standards, and credentials. Rapid tech changes, however, have created instability for the economy and society.

## Data Availability

Not applicable.
